# *QuickStats*: Percentage* of Adults Aged ≥20 Years Who Reported Being Told by a Doctor or Health Professional to Increase Their Physical Activity,^†^ by Age Group and Obesity Status^§^ — National Health and Nutrition Examination Survey, United States, 2011–2014

**DOI:** 10.15585/mmwr.mm6642a11

**Published:** 2017-10-27

**Figure:**
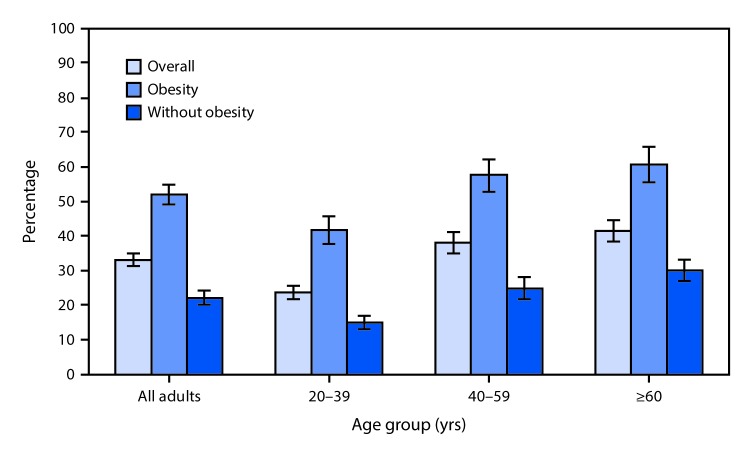
During 2011–2014, 33.2% of adults aged ≥20 years reported that a doctor or health professional told them to increase their physical activity. More than half (52.2%) of adults aged ≥20 years with obesity reported that a doctor or health professional told them to increase their physical activity compared with less than a quarter (22.3%) of adults without obesity. This pattern remained the same for all age groups examined. For both adults with and without obesity, the proportion who reported being told to increase their physical activity increased with age.

**Source:** National Center for Health Statistics, National Health and Nutrition Examination Survey, 2011–2014. https://www.cdc.gov/nchs/nhanes.htm.

